# Comparing Intraoperative Blood Loss in Cemented, Uncemented, and Hybrid Total Hip Replacement for Neck of Femur Fractures

**DOI:** 10.7759/cureus.42498

**Published:** 2023-07-26

**Authors:** Syed Haque, Abubakar Mustafa, Gopal Krishna Verma, Waqar Saadat, Emeka Okoye, Poornanand Goru, Mobeen Ismail

**Affiliations:** 1 Trauma and Orthopaedics, Manchester University NHS Foundation Trust, Manchester, GBR

**Keywords:** intraoperative, hybrid, uncemented, cemented, fracture neck of femur, total hip replacement, blood loss

## Abstract

Background

As the aging population increases, osteoporotic neck of femur fracture cases will continue to rise. Although hemiarthroplasty or half hip replacement is the treatment of choice in a majority of patients, a small but definite cohort of patients would need a total hip replacement. In these elderly patients who often have comorbidities, the use of cement to fix the prosthesis is often quoted as beneficial in view of perceived lower blood loss compared to uncemented fixation of the prosthesis. However, the cementation of the implant in itself has inherent problems. This study examined three modalities of fixation of a prosthesis for total hip replacement in the neck of femur fractures, namely, cemented, hybrid, and uncemented, and compared their relative intraoperative blood loss.

Methodology

This is a retrospective study with a follow-up of two years. Patients who presented to a level 1 trauma center in an inner city metropolitan with neck of femur fractures and were treated by total hip replacement were included in the study. Intraoperative blood loss was calculated using Nadler’s formula.

Results

There was no statistical difference in intraoperative blood loss in either of the three groups of patients, namely, cemented, hybrid, or uncemented total hip replacement for neck of femur fractures.

Conclusions

Intraoperative blood loss should not influence the modality of prosthesis fixation for total hip replacement in neck of femur fractures.

## Introduction

A neck of femur fracture (NOF) in the elderly is associated with significant morbidity and mortality. Approximately 1.6 million hip fractures occur worldwide each year, and by 2050, this number is expected to reach between 4.5 million and 6.3 million [1]. Around 76,000 patients are admitted with NOF in the United Kingdom every year [2]. Nearly half of these sustain displaced intracapsular fractures [3]. The National Institute for Health and Care Excellence (NICE), United Kingdom has produced guidance (Clinical Guidance 124) for the management of these fractures. Regarding surgical intervention for these fractures, it suggests performing replacement arthroplasty either hemiarthroplasty or total hip replacement (THR) in patients with displaced intracapsular NOF. The guideline further recommends offering THR in patients who are able to walk independently outdoors with no more than the use of a stick, are not cognitively impaired, and are medically fit for anesthesia and the procedure [4].

Hemiarthroplasty in the past and still is the most common operation performed in elderly patients with intracapsular NOF [5]. Cemented hemiarthroplasty has been preferred and recommended by NICE over uncemented implants as various studies have shown better outcomes with cemented implants including less pain and less deterioration in mobility in the long term [6]. Compared to cemented hemiarthroplasty uncemented hemiarthroplasty is also associated with a higher incidence of aseptic loosening and intraoperative femoral shaft fracture [7]. There is a common assumption among orthopedic surgeons that the application of cement plugs the holes created by rasping the medullary canal; hence, the cemented femoral component would bleed less and lead to less intraoperative blood loss compared to uncemented THR [8]. However, studies have not been able to prove this assumption. On the contrary, some studies have suggested less intraoperative blood loss in uncemented hemiarthroplasty compared to cemented hemiarthroplasty of the hip for NOF surgery [9].

Compared to hemiarthroplasty of the hip for NOF, THR is associated with significantly more intraoperative blood loss [10]. Significant blood loss and transfusion are clinically relevant and have prognostic value in patients undergoing major surgery [11]. Excessive blood loss is strongly associated with in-hospital mortality and morbidity [12].

THR has two components that have an interface with the bone, i.e., the acetabular cup and the femoral stem. Depending on whether we cement either both or none, THR can be either cemented, hybrid, or uncemented. In the hybrid component, the preferential method is to cement the femoral stem and the cup is uncemented; however, this can be done in a reverse manner as well and hence the name reverse hybrid.

No study has examined blood loss in patients who underwent THR for the management of NOF comparing all three types of THR, namely, cemented, uncemented, and hybrid THR. This study aimed to compare the perioperative blood loss for NOF patients who underwent all three types of fixations for THR, i.e., cemented, hybrid, and uncemented.

## Materials and methods

This study was conducted in a major trauma center. The hospital admits around 250 NOF patients every year. This is one of the few centers in the United Kingdom where more than 50% of patients eligible for THR as per NICE guidelines underwent THR [13]. Data were collected retrospectively from the electronic records database and clinical notes. The operations were done by lower limb arthroplasty surgeons who regularly perform THR in elective settings.

The inclusion criteria were patients aged above 60 years who had sustained NOF and were managed with a THR. Patients with NOFs who were less than 60 years of age, those with intracapsular NOF which was treated by internal fixation, and those who had reverse hybrid THR, i.e., the acetabular component was cemented, and the femoral component was uncemented, were excluded. The reverse hybrid hip replacement was excluded as this is rarely used. In our study, two patients had undergone reverse hybrid hip replacement. Patients included in this study had undergone THR between February 2017 and April 2019. They were consecutive patients. Due to the size of the sample, study power was not done.

The procedures were performed under either spinal or general anesthesia which was agreed upon by the anesthetist and the patients. Depending on the surgeon’s choice, hip replacement was performed in the lateral or supine position. Wounds were closed in layers, and skin clips were used to close the skin. Postoperatively, patients were given standard physiotherapy.

The primary outcome was total blood loss, determined from the difference between the preoperative hematocrit and the lowest postoperative hematocrit levels during hospitalization or the lowest postoperative hematocrit level before blood transfusion [14]. Nadler’s formula was used for calculating the blood column [15]. Once the blood column was calculated, blood loss was calculated by using the following formula: blood loss = preoperative blood volume × [In preop HCT - In postop HCT (1 + 0.15 × blood volume change) [16]. Postoperatively, all patients were given low-molecular-weight heparin as per NICE guidelines. Intraoperatively, patients were given tranexamic acid depending on the surgeon’s preference.

## Results

A total of 62 patients who underwent THR for NOF were included in the study. Among these, 41 were females and 21 were males, with their age ranging from 60 years to 91 years. Perioperative blood loss was calculated using the formulas of Gross and Nadler [16].

Patients were divided into three groups depending on the use of cement for implant fixation. The first group included 19 patients who had both the acetabular and femoral component cemented. The second group was the hybrid group, i.e., the acetabular component was uncemented and the femoral component was cemented. A total of 22 hybrid hip replacements were included in the study. The third and final group of patients were those in whom cement was not used so both the acetabular and femoral components were fixed and uncemented. A total of 21 uncemented THRs were included in the study.

The perioperative blood loss in cemented THR for NOF ranged between 0.11 L to 1.79 L with a median of 1.08 L. Similarly, in hybrid THR for NOF, the range was from 0.69 L to 2.58 L with a median of 0.92 L. For uncemented THR for NOF, the blood loss ranged between 0.152 L and 2.06 L with a median of 0.92 L (Table 1).

**Table 1:** Perioperative blood loss in the three groups.

Group	N	Mean	SD	Median	IQR	Minimum	Maximum
1 (Cemented)	19	1.1086	0.4864	1.0803	0.8692–1.5171	0.1152	1.7996
2 (Hybrid)	22	1.07110	0.64237	0.92076	0.63892–1.59120	0.06949	2.58810
3 (Uncemented)	21	0.9012	0.4808	0.9272	0.5473–1.1894	0.1525	2.0684

**Figure 1:**
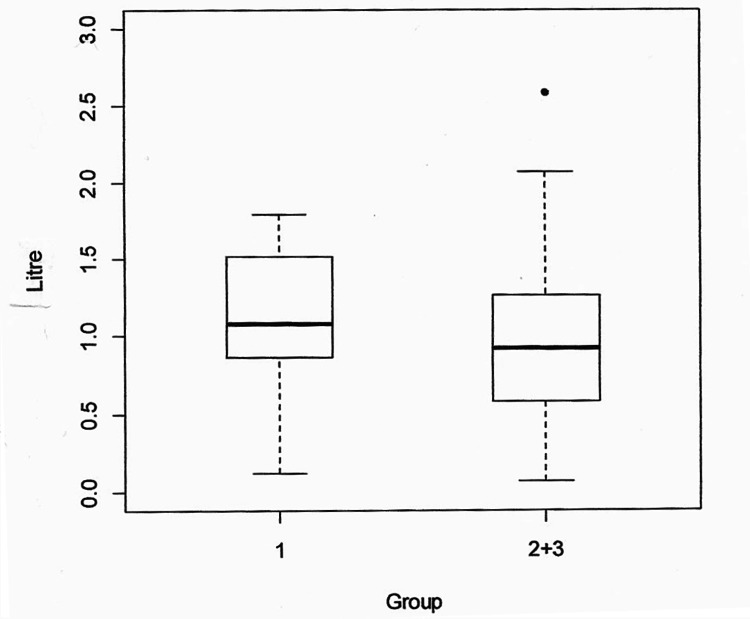
Comparison of perioperative blood loss between group 1 (cemented) and group 2 (hybrid) with group 3 (uncemented).

When comparing perioperative blood loss in the cemented THR group with the hybrid and uncemented THR group using a t-test, a non-significant difference was found with a p-value of 0.3991 (Figure 1).

**Figure 2:**
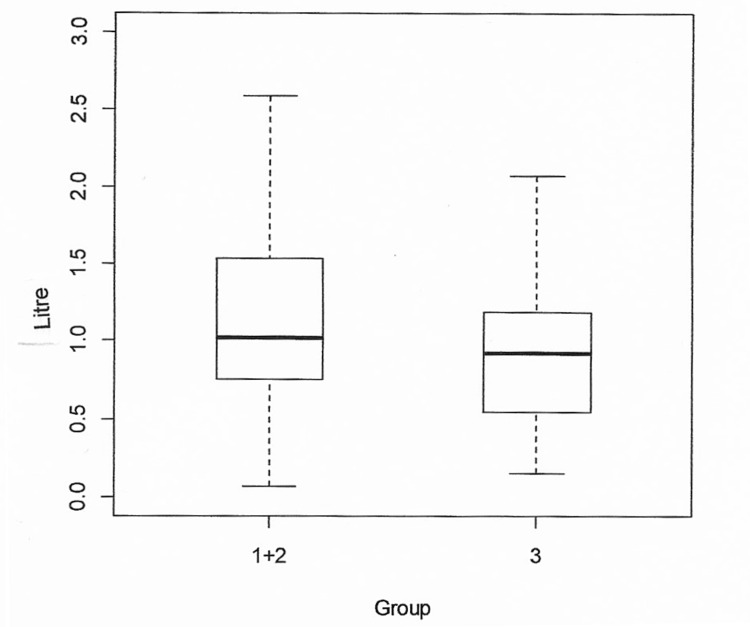
Comparison of perioperative blood loss between group 1 (cemented) and group 2 (hybrid) with group 3 (uncemented).

On comparing perioperative blood loss considering the cemented and hybrid group to the uncemented group, there was no significant statistical difference with a p-value of 0.1795 (Figure 2).

## Discussion

The number of hip fractures is expected to rise exponentially worldwide and is expected to be 2.5 million by 2050 [17]. Following NICE guidelines [2], there has been an increasing trend to perform THR in eligible patients with NOF [18]. According to the UK National Joint Registry’s most recent data, the most common mode of fixation of THR for NOF was hybrid fixation, followed closely by cemented fixation. Uncemented fixation is the least common, with only one in seven THRs for NOF being uncemented [18].

Hemorrhage is the number one killer in the operating room globally [19]. Significant preoperative blood loss can result in severe hypotension, and severe hypoxemia which can trigger cardiac arrest or permanent disability of the central nervous system. There is significant blood loss in THR which in elderly medically compromised patients makes them even more vulnerable to complications [20].

There is continued debate as to whether cemented or cementless implants should be utilized for THR in NOF. Literature is ambiguous in this. A recent study considering the most updated annual reports from five international joint arthroplasty registries with more than five years of follow-up concluded that there is no convincing difference between either method of fixation [21]. Another study examined the cost benefits of patients above the age of 70 undergoing cemented and uncemented THR for NOF and found no difference [22].

Significant blood loss can lead to blood transfusion which has its own complications, including transfusion-related lung injury, immunomodulation, and transmission of pathogens. Studies have also indicated that blood transfusion increases the risk of early and late morbidity and mortality [20]. Further allogeneic blood transfusion is associated with a longer hospital stay in patients undergoing primary THR increasing the overall cost of treatment [23].

This study investigated blood loss in THR for NOF. Previous studies have proven that there is no difference in perioperative blood loss for cemented and uncemented primary THR for osteoarthritis (8). This study further adds to it and confirms that there is no statistical difference in blood loss for cemented, hybrid, and uncemented THR in patients with NOF.

This study has some limitations. The data were collected retrospectively. Moreover, the sample size in each cohort was small. One of the reasons for this was that the modern trend of performing hybrid THR for NOF [18] led to a small number of uncemented THR in the cohort. To match the numbers of uncemented THR, the number was reduced in the other two groups. The information obtained from this study is important in its ability to solidify the foundation from which blood management decisions can be made.

This study provides insight into a very significant aspect of the operative management of these medically challenging patients. We hope this would generate interest in clinicians to further explore blood loss in THR for NOF by conducting randomized control trials.

## Conclusions

This study does not show any difference in blood loss for cemented and uncemented THR for NOF. With a shorter operating time compared to cemented THR and an absence of risk of cementation-related complications, uncemented THR may be considered in patients with NOF. However, this needs to be further evaluated with a larger powered and multicenter study.

The decision to perform cemented, hybrid, or uncemented THR in NOF should be a shared care decision-making between the patient and the treating physician taking into account a multitude of factors such as bone quality, the longevity of the implant, patient life expectancy, and cement-related risk factors. Cemented implants perform better in elderly patients whereas uncemented THR in NOF lasts longer in younger patients. Uncemented femoral component has a higher risk of periprosthetic fracture.

This study suggests that blood loss during THR for NOF should not be a criterion for offering cemented or uncemented THR in patients with NOF.

## Appendices

The total blood loss was calculated by applying the Gross formula [15]:

Total blood loss = PBV × (Hctpre − Hctpost)/Hctave

PBV = predicted blood volume

Hctpre = the initial preoperative hematocrit level

Hctpost = the lowest postoperative hematocrit level during hospitalization or the lowest postoperative hematocrit prior to blood transfusion

Hctave = the average of the Hctpre and Hctpost

The PBV was assessed according to the formula of Nadler [16]:

PBV (mL) = k1 × height (m) + k2 × weight (kg) + k3; k1 = 0.3669, k2 = 0.03219, and k3 = 0.6041 for men; k1 = 0.3561, k2 = 0.03308, and k3 = 0.1833 for women. If a reinfusion or an allogeneic transfusion is performed, the volume transfused should be added when calculating total blood loss.

## References

[1] (2022). International Osteoporosis Foundation. Facts and statistics. https://www.iofbonehealth.org/facts-statistics.

[2] (2022). National Institute for Health and Care Excellence. Hip fracture: management. https://www.nice.org.uk/guidance/cg124/chapter/Recommendations.

[3] (2022). Will my surgeon offer the type of operation recommended by NICE? National Hip Fracture Database. Report.

[4] (2022). The National Institute for Health and Care Excellence (NICE). Surgical procedures, hip fracture management; CG124. https://www.nice.org.uk/guidance/cg124.

[5] Figved W, Opland V, Frihagen F, Jervidalo T, Madsen JE, Nordsletten L (2009). Cemented versus uncemented hemiarthroplasty for displaced femoral neck fractures. Clin Orthop Relat Res.

[6] Parker MI, Pryor G, Gurusamy K (2010). Cemented versus uncemented hemiarthroplasty for intracapsular hip fractures: a randomised controlled trial in 400 patients. J Bone Joint Surg Br.

[7] Okike K, Chan PH, Prentice HA, Paxton EW, Burri RA (2020). Association between uncemented vs cemented hemiarthroplasty and revision surgery among patients with hip fracture. JAMA.

[8] Trice ME, Walker RH, D'Lima DD, Morris BA, Colwell CW Jr (1999). Blood loss and transfusion rate in noncemented and cemented/hybrid total hip arthroplasty. Is there a difference? A comparison of 25 matched pairs. Orthopedics.

[9] Elmenshawy AF, Salem KH (2021). Cemented versus cementless bipolar hemiarthroplasty for femoral neck fractures in the elderly. EFORT Open Rev.

[10] Blomfeldt R, Törnkvist H, Eriksson K, Söderqvist A, Ponzer S, Tidermark J (2007). A randomised controlled trial comparing bipolar hemiarthroplasty with total hip replacement for displaced intracapsular fractures of the femoral neck in elderly patients. J Bone Joint Surg Br.

[11] Wu WC, Smith TS, Henderson WG (2010). Operative blood loss, blood transfusion, and 30-day mortality in older patients after major noncardiac surgery. Ann Surg.

[12] Karkouti K, Wijeysundera DN, Yau TM (2004). The independent association of massive blood loss with mortality in cardiac surgery. Transfusion.

[13] (2022). National Hip Fracture Database (NHFD) Charts and Reports. Dashboard report for Manchester Royal Infirmary. https://www.nhfd.co.uk/20/NHFDcharts.nsf/fmdashboard?readform&year=2019&org=MRI.

[14] Yue C, Kang P, Yang P, Xie J, Pei F (2014). Topical application of tranexamic acid in primary total hip arthroplasty: a randomized double-blind controlled trial. J Arthroplasty.

[15] Gross JB (1983). Estimating allowable blood loss: corrected for dilution. Anesthesiology.

[16] Nadler SB, Hidalgo JH, Bloch T (1962). Prediction of blood volume in normal human adults. Surgery.

[17] Cheung CL, Ang SB, Chadha M (2018). An updated hip fracture projection in Asia: the Asian Federation of Osteoporosis Societies study. Osteoporos Sarcopenia.

[18] (2022). National Joint Registry. Indications for primary hip procedures based on age groups. https://reports.njrcentre.org.uk/hips-primary-procedures-patient-characteristics/H07v1NJR.

[19] Irita K (2011). Risk and crisis management in intraoperative hemorrhage: human factors in hemorrhagic critical events. Korean J Anesthesiol.

[20] Carling MS, Jeppsson A, Eriksson BI, Brisby H (2015). Transfusions and blood loss in total hip and knee arthroplasty: a prospective observational study. J Orthop Surg Res.

[21] Zhang CF, Yan CH, Zhang WM (2017). Cemented or cementless fixation for primary hip arthroplasty - evidence from the International Joint Replacement Registries. Ann Joint.

[22] Marinelli M, Soccetti A, Panfoli N, de Palma L (2008). Cost-effectiveness of cemented versus cementless total hip arthroplasty. A Markov decision analysis based on implant cost. J Orthop Traumatol.

[23] Bou Monsef J, Boettner F (2014). Blood management may have an impact on length of stay after total hip arthroplasty. HSS J.

